# Cancer Patients First Treated with Chemotherapy: Are They More Likely to Receive Surgery in the Pandemic?

**DOI:** 10.3390/curroncol29100611

**Published:** 2022-10-14

**Authors:** Rui Fu, Rinku Sutradhar, Anna Dare, Qing Li, Timothy P. Hanna, Kelvin K. W. Chan, Jonathan C. Irish, Natalie Coburn, Julie Hallet, Simron Singh, Ambica Parmar, Craig C. Earle, Lauren Lapointe-Shaw, Monika K. Krzyzanowska, Antonio Finelli, Alexander V. Louie, Ian J. Witterick, Alyson Mahar, David R. Urbach, Daniel I. McIsaac, Danny Enepekides, Nicole J. Look Hong, Antoine Eskander

**Affiliations:** 1 ICES, Toronto, ON M4N 3M5, Canada; 2 Institute of Health Policy, Management, and Evaluation, University of Toronto, Toronto, ON M5T 3M6, Canada; 3 Department of Otolaryngology—Head and Neck Surgery, University of Toronto, Toronto, ON M5G 1X5, Canada; 4 Department of Surgery, University of Toronto, Toronto, ON M5T 1P5, Canada; 5 Division of Cancer Care and Epidemiology, Cancer Research Institute, Queen’s University, Kingston, ON K7L 3N6, Canada; 6 Ontario Institute for Cancer Research (OICR), Toronto, ON M5G 0A3, Canada; 7 Odette Cancer Centre—Sunnybrook Health Sciences Centre, Toronto, ON M4N 3M5, Canada; 8 Ontario Health—Cancer Care Ontario, Toronto, ON M5G 2L7, Canada; 9 Department of Otolaryngology—Head & Neck Surgery/Surgical Oncology, University of Toronto, Princess Margaret Cancer Centre, Toronto, ON M5G 1X5, Canada; 10 Department of Medicine, University of Toronto, Toronto, ON M5S 1A8, Canada; 11 Department of Radiation Oncology, University of Toronto, Toronto, ON M5T 1P5, Canada; 12 School of Nursing, Queen’s University, Kingston, ON K7L 3N6, Canada; 13 Department of Surgery, Women’s College Hospital, Toronto, ON M5S 1B2, Canada; 14 Department of Anesthesiology and Pain Medicine, The Ottawa Hospital, Ottawa, ON K1Y 4E9, Canada

**Keywords:** COVID-19, cancer, chemotherapy, cancer surgery, health inequity

## Abstract

Due to the ramping down of cancer surgery in early pandemic, many newly diagnosed patients received other treatments first. We aimed to quantify the pandemic-related shift in rate of surgery following chemotherapy. This is a retrospective population-based cohort study involving adults diagnosed with cancer between 3 January 2016 and 7 November 2020 in Ontario, Canada who received chemotherapy as first treatment within 6-months of diagnosis. Competing-risks regression models with interaction effects were used to quantify the association between COVID-19 period (receiving a cancer diagnosis before or on/after 15 March 2020) and receipt of surgical reSection 9-months after first chemotherapy. Among 51,653 patients, 8.5% (*n* = 19,558) of them ultimately underwent surgery 9-months after chemotherapy initiation. Receipt of surgery was higher during the pandemic than before (sHR 1.07, 95% CI 1.02–1.13). Material deprivation was independently associated with lower receipt of surgery (least vs. most deprived quintile: sHR 1.11, 95% CI 1.04–1.17), but did not change with the pandemic. The surgical rate increase was most pronounced for breast cancer (sHR 1.13, 95% CI 1.06–1.20). These pandemic-related shifts in cancer treatment requires further evaluations to understand the long-term consequences. Persistent material deprivation-related inequity in cancer surgical access needs to be addressed.

## 1. Introduction

### 1.1. Background

Surgery is central to the management of many cancers as it represents the only curative modality. The ongoing COVID-19 pandemic has caused disruption in cancer services with evidence of delayed diagnoses and poor access to surgery [1]. As surgeries were delayed, the use of neoadjuvant chemotherapy was expanded. The American College of Surgeons recommended using neoadjuvant therapy for eligible people with breast, colorectal or thoracic cancers as hospitals redirected resources toward the care of patients with COVID-19 [2]. The National Health Service established surgical hubs to operate on patients who urgently needed surgery in the next 24–72 h [3]. In Ontario, Canada, surgical priority was given to patients with life-threatening conditions and those with obstructed, perforated, or actively bleeding cancers [4,5].

### 1.2. Cancer Care Continuum and Inequity during the COVID-19 Pandemic

Whether such changes in therapeutic approaches impacted downstream patient treatment and outcomes is unclear [6]. More specifically, whether newly diagnosed patients receiving upfront chemotherapy went on to get surgery for cancer within a clinically reasonable time window is unknown. A literature search on MEDLINE revealed only 5 studies documenting the subsequent receipt of a cancer surgical resection among new cancer patients initially treated non-surgically during the COVID-19 pandemic (Appendix A) [7,8,9,10,11]. Notably, newly diagnosed oesophageal cancer patients in Ireland and breast cancer patients in Turkey did not experience a different rate of surgery following neoadjuvant therapy during the pandemic compared to their pre-pandemic counterparts [7,10]. However, in India, the receipt of surgery after neoadjuvant chemotherapy has dropped among ovarian cancer patients from 64% in 2019 to just 33% during 2020 [8]. Likewise, The Netherlands reported significantly prolonged wait times from neoadjuvant radiation or chemoradiation initiation to surgery in colorectal cancer patients in 2020 than in 2018/2019 [11]. The only real-world results that point to a clearly increased receipt of surgery following neoadjuvant therapy during the pandemic come from Canada; specifically, among newly diagnosed breast cancer patients who had been first treated by hormonal therapy within 6 months of diagnosis, their subsequent receipt of surgery was indeed higher during the pandemic period than before, although such difference was not observed among those initially managed by chemotherapy [9]. Overall, these conflicting results suggest a lack of clear understanding on how cancer care continuum has been shifted by the pandemic. The strength of evidence is further limited by the small sample size of existing studies and their focus on a single disease site. As such, there is a need to identify if the pandemic has hindered the sequence of cancer care beyond its apparent impact on the provision of individual cancer treatment modality. These insights on the degree to which the healthcare system was able to maintain the delivery of standard cancer care services under a rapidly evolving, resource-intensive public health emergency will provide guidance on how to care for these cancer patients in the long term and how to adjust the model of care to prepare the system for the next health catastrophe [12]. 

It is also imperative to examine if the pandemic-related disruptions on cancer care continuum and their impact were inequitably distributed, and how this related to demography, geography, and social determinants of health. Inequities in healthcare access and outcomes can be accentuated along the continuum of care as patients progress from diagnosis to first and then subsequent treatment modalities, a phenomenon that has been described in low socioeconomic status (SES), rural dwelling, and racialized populations [13]. As such, cancer patients with low SES may have faced additional barriers during the pandemic in accessing surgery after chemotherapy, a hypothesis yet to be formally assessed [6,9,14].

The two-fold objective of this study was (1) to quantify the impact of the pandemic on surgical resection rates for patients receiving chemotherapy as their first cancer treatment and (2) to examine whether surgery rates following chemotherapy differed by SES.

## 2. Materials and Methods

### 2.1. Study Design

This retrospective population-based cohort study was conducted in Ontario, Canada where 14.6 million permanent residents have universal access to physician services under the Ontario Health Insurance Plan (OHIP) [15]. Administrative datasets were linked using unique encoded identifiers and analyzed at ICES, formerly known as the Institute for Clinical Evaluative Sciences, a prescribed entity under Ontario’s Personal Health Information Protection Act (PHIPA). Section 45 of PHIPA authorizes ICES to collect personal health information without consent and allows research projects that use these data to be exempt from review by a Research Ethics Board. The use of data in this study was approved by ICES’ Privacy and Legal Office.

### 2.2. Data Sources

The Ontario Cancer Registry (OCR) captures 96% of index cancers across the province [16,17]. The Registered Persons Database (RPDB) includes sociodemographic and vital status data of permanent residents. The Immigration, Refugees and Citizenship Canada (IRCC) Permanent Resident Database (with data from January 1985 to May 2017) includes records of individuals who immigrated to Ontario during this period. The OHIP claims database contains physician billing records for chemotherapy visits. Canadian Institute for Health Information (CIHI)’s Hospital Discharge Abstract Database and Same-day Surgery Database contain information on surgical procedures performed at a hospital. Statistics Canada’s Postal Code Conversion File (PCCF) contains the status of rural living [18]. The Ontario Marginalization Index (ONMARG) database stores information on material deprivation, a validated measure of SES that encompasses the family structure, living condition, education, employment, income, and receipt of government transfer payments in a neighborhood [19].

### 2.3. Study Cohort

We identified adults (age 18 or above) who were newly diagnosed with cancer between 3 January 2016 and 7 November 2020 and received chemotherapy within 6 months of diagnosis as their first cancer treatment. The accrual end date (November 2020) reflected the last update of cancer incidence data from the OCR at time of this analysis (January 2022). If multiple cancer diagnoses occurred during the study period, only the earliest diagnosis was selected. We excluded patients with a diagnosis of melanoma or skin cancer to ensure a robust capture of at-hospital cancer surgeries given these are usually treated in the outpatient setting; patients with cancers primarily labelled as ophthalmologic and paraneoplastic neurological syndromes were also excluded due to rarity (<0.04% of the cohort). Patients were followed from first date of chemotherapy for 9 months, or to date of surgery, or until date of death, or to end of study (26 June 2021), whichever occurred first. Due to the lag of data in databases that specifically maintain records of chemotherapy visits (the Cancer Activity Level Reporting and the Ontario Drug Benefit databases) at time of this analysis, we restricted chemotherapy to be physician-supervised intravenous infusions billable using the OHIP G-codes [15]. Hence, oral drugs including endocrine therapy for breast and prostate cancers were not identified.

### 2.4. Outcome—Time to Surgery after First Chemotherapy

We defined our outcome to be the time (measured in months) from the first date of chemotherapy to the date of surgery. Patients who were alive without getting surgery at the end of the follow-up window were censored. Surgical codes were obtained from CIHI and ascertained using the OCR diagnosis records to ensure a match between surgical site and cancer type. We also ensured that only resections, not biopsies, were captured.

### 2.5. Exposure—COVID-19 Pandemic Time Period

The main exposure was whether cancer diagnosis occurred before or after the start of the COVID-19 pandemic using 15 March 2020 when hospitals were advised to cancel nonemergent and elective procedures by the province’s Chief Medical Officer of Health to represent the start date of COVID-19 [20]. The period before this date (from 3 January 2016 to 14 March 2020) was termed pre-pandemic and the period thereafter (from 15 March 2020 to 7 November 2020) was the pandemic period [21]. A binary variable was created using pre-pandemic as the reference level.

### 2.6. Covariates

We considered sociodemographic and clinical characteristics of patients at time of cancer diagnosis (baseline). Patient age and sex were obtained from the RPDB. Rural living, defined as living in a rural area or a small town with a population of less than 10,000, was extracted from the PCCF [18]. Immigration status was identified from the IRCC Permanent Resident Database. Material deprivation from the ONMARG was reported in quintiles [19]. Comorbidity was measured by the Elixhauser Comorbidity Index using hospitalization records over the past 5 years [22]. Five comorbidity groups were created for patients scored 0, 1, 2, 3+ on the index and for those who were not hospitalized [23,24]. Cancer type was determined using the OCR diagnosis records.

### 2.7. Statistical Analysis

Descriptive analyses were carried out to compare the baseline characteristics of patients by COVID-19 period using 0.10 as a standardized difference threshold to identify a significant difference [25]. For the sub-cohort that received a surgical resection during the follow-up, we used the t-test and the Wilcoxon Rank-Sum test to compare their mean and median time spent from first chemotherapy to surgery by COVID-19 period.

For the entire cohort, we studied time to surgery after first chemotherapy using a time-to-event analytical framework, with all-cause death being modelled as a competing risk of receiving surgery [26]. We estimated the cumulative incidence functions (CIFs) of surgery by COVID-19 period and compared them using the Gray’s Test [27]. To quantify the association between COVID-19 period and time to surgery after first chemotherapy, we constructed four Fine-Gray subdistribution hazards models [28]: the first model was univariable with only the COVID-19 period exposure variable; the second model was multivariable and accounted for all a priori chosen patient characteristics; the third model added interaction terms between the COVID-19 period and each cancer type to the second model to assess if the pandemic has impacted cancer types differently. The final model included interaction terms between COVID-19 period and material deprivation quintile to the second model to test if surgical inequalities were accentuated by the pandemic. All analyses were 2-sided using *p*-value < 0.05 to identify statistical significance. Analyses were performed on SAS Enterprise Guide 7.15 (SAS Institute, Inc., Cary, NC, USA).

## 3. Results

Our cohort included 51,653 adults diagnosed with cancer between 3 January 2016 and 7 November 2020 and who received chemotherapy as the first cancer treatment within 6 months of diagnosis (Table 1). Compared with patients diagnosed in the pre-pandemic period (*N* = 45,807, 88.7%), those who received the diagnosis afterwards (*N* = 5846, 11.3%) were more likely to have breast cancer (19.4% vs. 14.6%, standardized difference 0.13) and started chemotherapy faster after diagnosis (mean days ± SD, 38.44 ± 30.62 vs. 43.69 ± 34.11; standardized difference 0.16). The mean follow-up duration after first chemotherapy was 7.06 ± 2.79 months and 6.69 ± 2.76 months, respectively, for those diagnosed in pre-pandemic and the pandemic period (standardized difference 0.13).

Nine months after first chemotherapy, 18.5% (*N* = 9558) of patients underwent a surgical resection. Among them, the median time from first chemotherapy to surgery was 4.31 months, regardless of whether the cancer was diagnosed in pre-pandemic (interquartile range (IQR) 3.19–4.96) or during the pandemic (IQR 3.22–4.90, *p*-value 0.93). The mean time to surgery was also identical between the two groups (4.10 ± 1.64 vs. 4.09 ± 1.62 months, *p*-value 0.74).

**Figure 1:**
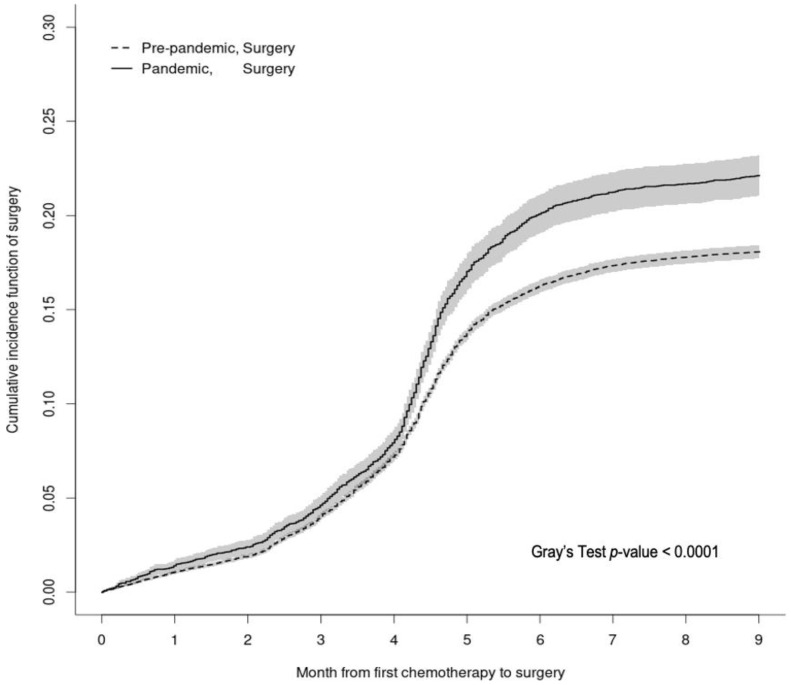
Cumulative Incidence functions of surgery received within 9-month after first chemotherapy for patients first treated with chemotherapy by COVID-19 period. Legend: Death (not shown) was modelled as a competing-risk event. The pre-pandemic period (dashed) is from 3 January 2016 to 14 March 2020, while the pandemic period (solid) is from 15 March 2020 to 7 November 2020. At each time point after the date of first chemotherapy, the cumulative incidence of receiving surgery was higher in the pandemic period than in pre-pandemic (Gray’s *p*-value < 0.0001).

Figure 1 shows the CIFs of receiving surgery within 9 months after first chemotherapy, treating death as a competing risk. Patients who were diagnosed with cancer during the pandemic were significantly more likely to receive surgery after first chemotherapy, especially after the 4.5th month (Gray’s test *p*-value < 0.01). Specifically, 4 months after first chemotherapy, the probability of receiving surgery was 7.9% (95% confidence interval [CI] 7.2%–8.6%) among patients diagnosed during the pandemic compared to 7.2% (95% CI 6.9%-7.4%) for those diagnosed in the pre-pandemic period. The difference grew to 20.1% (95% CI 19.1%–21.1%) vs. 16.3% (95% CI 15.9%–16.6%) 6 months after first chemotherapy, and further escalated to 22.1% (95% CI 21.1%–23.2%) vs. 18.1% (95% CI 17.7%–18.4%) at 9-months.

In Table 2, we report that in the univariable model, the rate of surgery among those who received chemotherapy as initial cancer treatment increased in the pandemic relative to pre-pandemic period (subdistribution hazard ratio (sHR) 1.25, 95% CI 1.18–1.32). After adjusting for covariates, surgery use remained higher for those diagnosed during the pandemic (sHR 1.07, 95% CI 1.02–1.13). Additional factors that were independently associated with increased receipt of surgery were younger age, the least deprived quintile, lower comorbidity, and having breast cancer. Notably, patients in the least deprived quintile (representing the highest SES) were more likely to receive surgery (sHR 1.11, 95% CI 1.04–1.17) than those in the most deprived quintile.

**Figure 2:**
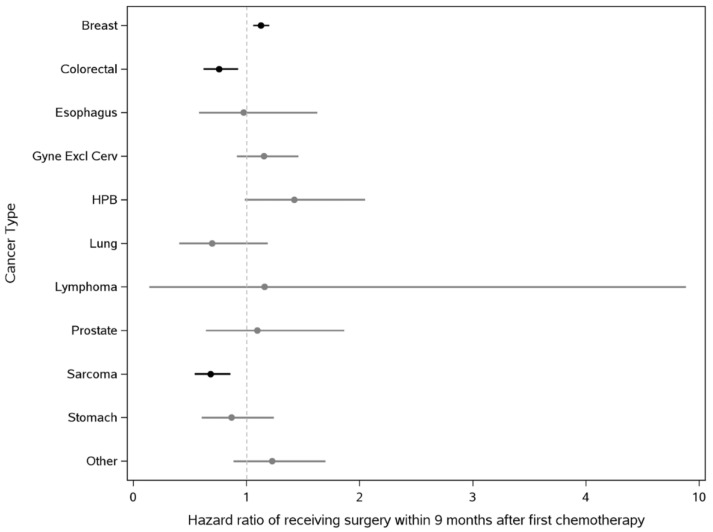
The COVID-19 pandemic impact on the use of surgery after chemotherapy initiation by cancer type. Legend: We report the subdistribution hazard ratios (sHRs) and associated 95% confidence intervals of receiving surgery within 9 months after chemotherapy initiation in the pandemic relative to pre-pandemic period for each cancer type. sHRs were computed from a multivariable Fine-Gray regression model, where interaction of the COVID-19 pandemic indicator (pandemic vs. pre-pandemic) with cancer type was included. “Other” includes central nervous system, cervical, endocrine, genitourinary, head and neck, and other cancer types with unknown or ill-defined primary site. sHRs that are significantly different from 1 were highlighted in black, while insignificant sHRs were plotted in gray. A sHR > 1 implies an increased use of surgery within 9-month following chemotherapy initiation in the pandemic relative to pre-pandemic period.

By adding interaction terms between the COVID-19 period and cancer type to the multivariable model (Figure 2), we found the pandemic impact on surgery use to differ by cancer type overall (Type-III p-value of interaction terms < 0.01) and that individually, the difference was significant for breast, colorectal, and sarcoma cancers (*p*-value of each interaction term < 0.01). In the pandemic period, the receipt of surgery for breast cancer increased (sHR 1.13, 95% CI 1.06–1.20) following chemotherapy as first treatment, whereas that of colorectal (sHR 0.76, 95% CI 0.63–0.92) and sarcoma (sHR 0.69, 95% CI 0.55–0.86) cancers both decreased. Examination of interaction terms between the COVID-19 period and material deprivation revealed an absence of accentuated surgical access inequalities in the pandemic, as the interaction effect was found insignificant (Type-III *p*-value of interaction terms 0.37).

## 4. Discussion

In our cohort of 51,653 patients who were first treated with chemotherapy within 6 months of diagnosis, their subsequent receipt of surgery was significantly higher in the pandemic than before. People with breast cancer exhibited the most pronounced increase in subsequent surgical use. Material deprivation was independently and negatively associated with surgery use after first chemotherapy and there were no changes in the pandemic.

There are several potential reasons for our observations. The increased rate of surgery during the pandemic period after chemotherapy might reflect patients who would have otherwise had surgery upfront but were placed on neoadjuvant chemotherapy instead due to directed prioritization of non-surgical cancer treatments at the beginning of the pandemic [4,5]. This speculation is supported by a range of population-level analyses showing a sharp decrease in cancer surgery volume in the early pandemic [1,23,29,30]. Our results may also reflect the temporal shifts towards neoadjuvant approaches that are not specifically driven by the pandemic [31,32]. Stage migration due to the abrupt cessation of screening and disruptions in other cancer services could have also led to higher use of chemotherapy in both neoadjuvant and palliative settings [33]. Because stage data is not yet available in our cancer registry for the pandemic period, future study with this data can confirm whether the higher rate of surgery observed reflects the pandemic impact on patients’ disease status and prognosis or represents a deviation from standard cancer therapy. These insights are needed to formulate treatment plans to maximize long-term patient outcomes.

In line with a recently published report [9], we found breast cancer patients receiving chemotherapy as the first treatment to have the most significant increase in subsequent surgery use during the pandemic. Incident detection of breast cancer was severely hindered by COVID-19 since cancer screening including mammograms for asymptomatic women was one of the most affected cancer care domains [34]. In Ontario during the first 6 months of COVID-19 alone, there were nearly 2000 less breast cancer diagnoses [24]. Further to the lowered incidence, single-centre data has shown a higher percentage of late-stage presentation of breast cancer during the pandemic than before [35,36], with some evidence suggesting a change in the distribution of subtypes [37]. For certain subtypes such as estrogen/progesterone receptor-positive cancers there has been an expanded use of molecular testing (such as Oncotype DX) on diagnostic core biopsies in recent years [38]. Collectively, these shifts have led to more use of neoadjuvant chemotherapy for breast cancer management during the pandemic [9,36], and, according to our results, more use of breast surgery 9 months after first chemotherapy as well. Further work will be required to demonstrate if 9-months represents a reasonable gap between neoadjuvant chemotherapy initiation and surgery. Modeling studies that aim to forecast the pandemic impact on breast cancer outcomes need to incorporate our data to improve the prediction accuracy [39,40,41].

We found surgical use following chemotherapy as the first treatment to only decrease for two cancer types, including sarcoma cancer which was rare (2% of our cohort). These findings may demonstrate the success of de-escalation measures to maintain cancer treatment [4,5]. Specifically, by employing chemotherapy as a mitigating tactic, most eligible patients were able to receive surgery after a reasonable delay that was not significantly greater than pre-pandemic levels. This also creates a serendipitous natural experiment where some patients received upfront surgery (pre-pandemic) while others received neoadjuvant treatment followed by surgery. Once stage data becomes available, real-world comparisons of these two approaches may be performed. For cancer types that are not typically managed by a neoadjuvant approach (such as prostate and lymphoma cancers), our results showed that for newly diagnosed patients who had indeed started first-line chemotherapy, they were equally likely to get surgery in the next 9 months during the pandemic and in pre-pandemic. With stage data future research can advance this finding by distinguishing palliative and neoadjuvant chemotherapy in these cancer types to better map out the pandemic impact on the treatment pathway for patients needing surgery.

Inequalities in care are noted in our data. We found material deprivation, a measure of lower SES, to be associated with lower use of surgery after first chemotherapy throughout the study period, and the magnitude of such inequality was the same during the pandemic. It is important to note that our results do not imply that patients of lower SES did not experience additional obstacles in receiving cancer care during the pandemic; on the contrary, many support resources such as transportation services and social/finance assistances were diminished, particularly early on. Cancer patients with lower SES reported struggling to navigate the complex medical environment amid the pandemic, including to adamantly advocate for having the standard curative treatment (surgery) [14]. It is possible that the observed absence of any change in inequality reflects a shift in incident patient profile in the pandemic, as those with lower SES were either not diagnosed at all or had higher rates of presenting with unresectable advance disease. With stage data future research can identify surgical resection candidates to delineate the inequity related to early cancer diagnosis (including poor access to primary care) from the inequity on surgical use after chemotherapy.

Our findings are subject to limitations. First, while we focused on patient-level factors in this analysis, access to cancer surgery is also impacted by health system capacity and hospital characteristics (structures of care) [12]. Future work should use a mixed-effect modeling strategy or an ecological study design to assess how the pandemic-related high-level shifts in the health system translates to individual-level differences in cancer surgery use. Next, we were unable to capture hormonal therapy, a procedure not explicitly identified in the physician billing data (OHIP) we used to establish chemotherapy visits. Future study needs to assess the surgical status among neoadjuvant hormonal therapy recipients to see if the pandemic has impacted them uniquely [9]. We also did not have data on cancer staging, and thereby, were unable to control for staging as a covariate in the regression analysis or distinguish chemotherapy used for palliative and neoadjuvant purposes. Finally, alternative indices could be used to pinpoint which dimension of SES limits the access to cancer surgery. Ideally, such an index needs to include race/ethnicity, which we could not adjust for in this analysis [42].

## 5. Conclusions

This study examined newly diagnosed cancer patients first treated by chemotherapy within 6 months of cancer diagnosis and found their use of surgery 9 months after first chemotherapy to be significantly higher after the start of COVID-19. This increase was the most significant in breast cancer. Further study should determine if the higher rate of surgery is attributed to a shift in patient profile or represents a deviation from the standard therapy. Material deprivation is associated with a reduced utilization of cancer surgery, which calls for more research to pinpoint the phase of cancer care where the inequity occurs. The findings of this study reveal major limitations in the current cancer system that must made significant and potentially harmful shifts in non-COVID-19 patient management during a public health catastrophe. There is an urgent need to expand system capacity to prepare for future emergency surges in care demand.

## Figures and Tables

**Table 1:** Characteristics of patients receiving chemotherapy as the first cancer treatment within 6 months of diagnosis, stratified by COVID-19 period (*n* = 51,653).

Characteristics	Cancer Diagnosed in Pre-Pandemic (*N* = 45,807, 88.7%)	Cancer Diagnosed in the Pandemic (*N* = 5846, 11.3%)	Standardized Difference ^a^
Age at diagnosis (Mean ± SD)	63.80 ± 14.27	63.46 ± 14.24	0.02
Female	24,066 (52.5%)	3234 (55.3%)	0.06
Rural residents	5591 (12.2%)	779 (13.3%)	0.03
Immigrants	6086 (13.3%)	839 (14.4%)	0.03
Material deprivation quintile ^b^			
1, least deprived	9990 (21.8%)	1258 (21.5%)	0.01
2	9534 (20.8%)	1275 (21.8%)	0.02
3	8791 (19.2%)	1191 (20.4%)	0.03
4	8698 (19.0%)	1052 (18.0%)	0.03
5, most deprived	8413 (18.4%)	1022 (17.5%)	0.02
Region			
Toronto	3678 (8.0%)	454 (7.8%)	0.01
Central	13,514 (29.5%)	1732 (29.6%)	0
East	11,529 (25.2%)	1510 (25.8%)	0.02
North	3075 (6.7%)	434 (7.4%)	0.03
West	14,010 (30.6%)	1716 (29.4%)	0.03
Comorbidity ^c^			
0	5384 (11.8%)	671 (11.5%)	0.01
1	3546 (7.7%)	435 (7.4%)	0.01
2	2240 (4.9%)	248 (4.2%)	0.03
3+	2790 (6.1%)	300 (5.1%)	0.04
No hospitalization	31,847 (69.5%)	4192 (71.7%)	0.05
Cancer type			
Breast	6682 (14.6%)	1137 (19.4%)	0.13
Central nervous system	172 (0.4%)	23 (0.4%)	0
Cervical	170 (0.4%)	15 (0.3%)	0.02
Colorectal	3,668 (8.0%)	453 (7.7%)	0.01
Endocrine	80 (0.2%)	11 (0.2%)	0
Esophagus	518 (1.1%)	70 (1.2%)	0.01
Genitourinary	766 (1.7%)	104 (1.8%)	0.01
Gynecologic (excluding cervical)	1760 (3.8%)	225 (3.8%)	0
Head and neck	522 (1.1%)	80 (1.4%)	0.02
Hepato-pancreatic biliary	3460 (7.6%)	490 (8.4%)	0.03
Lung	6222 (13.6%)	761 (13.0%)	0.02
Lymphoma	8293 (18.1%)	899 (15.4%)	0.07
Prostate	1447 (3.2%)	193 (3.3%)	0.01
Sarcoma	923 (2.0%)	101 (1.7%)	0.02
Stomach	1532 (3.3%)	161 (2.8%)	0.03
Other	9592 (20.9%)	1123 (19.2%)	0.04

^a^ We used 0.1 to identify a statistically and clinically significant imbalance in the distribution of characteristics between the two groups of patients. There were missing data for rural living status (0.2%) and material deprivation status (0.8%) that did not differ between the two patient groups (both standardized differences < 0.01). These patients were excluded from the multivariable regression analysis. ^b^ We measured material deprivation using the Ontario Marginalization Index that considers the following neighborhood characteristics: population that is without a high school diploma, unemployed, or considered low-income, and households that are single-parent families, receiving government transfer payments, or living in dwellings that need major repair. ^c^ The Elixhauser comorbidity index was computed using a 5-year look-back window to capture records of hospitalization in administrative databases.

**Table 2:** Competing risks regression results showing the COVID-19 impact on receipt of surgery in 9 months after first chemotherapy for patients first treated with chemotherapy.

Variables	sHR	95% CI	*p*-Value
**Univariable model**			
Cancer diagnosis in the pandemic vs. pre-pandemic ^a^	1.25	1.18–1.32	<0.01
**Multivariable model**			
Cancer diagnosis in the pandemic vs. pre-pandemic	1.07	1.02–1.13	0.01
Age at cancer diagnosis, each 10-year increase	0.83	0.82–0.84	<0.01
Females vs. males	1.00	0.92–1.08	0.93
Immigrants vs. non-immigrants	1.04	0.99–1.08	0.14
Material deprivation (vs. 5—most deprived) ^b^			
1—Least deprived	1.11	1.04–1.17	<0.01
2	1.06	1.00–1.13	0.06
3	1.03	0.96–1.09	0.43
4	1.00	0.94–1.07	0.97
Comorbidity (vs. no hospitalization)			
0	0.88	0.83–0.93	<0.01
1	0.84	0.76–0.93	<0.01
2	0.83	0.73–0.95	<0.01
3+	0.64	0.55–0.74	<0.01
Cancer type (vs. breast cancer)			
Colorectal	0.36	0.34–0.39	<0.01
Esophagus	0.38	0.32–0.45	<0.01
Gynecologic excluding cervical	0.90	0.82–0.97	<0.01
Hepato-pancreatic biliary	0.06	0.05–0.07	<0.01
Lung	0.031	0.026–0.036	<0.01
Lymphoma	0.001	0.000–0.002	<0.01
Prostate	0.10	0.08–0.12	<0.01
Sarcoma	0.000	0–0	<0.01
Stomach	0.33	0.29–0.37	<0.01
Other ^c^	0.028	0.025–0.031	<0.01

^a^ The pre-pandemic period is from 3 January 2016 to 14 March 2020, and the pandemic period is from 15 March 2020 to 7 November 2020. We verified the proportional-hazard assumption by examining an interaction term between the COVID-19 period and a binary variable denoting time since first chemotherapy > 4.5 months. Because this interaction term was insignificant (*p*-value 0.70), we ruled out any violation of the proportional-hazard assumption; ^b^ There was an overall negative association between material deprivation and time to surgery within 9-month after first chemotherapy (Type-III *p*-value < 0.01); ^c^ “Other” includes central nervous system, cervical, endocrine, genitourinary, head and neck, and other cancer types with unknown or ill-defined primary site.

## Data Availability

The dataset from this study is held securely in coded form at ICES. While legal data sharing agreements between ICES and data providers prohibit ICES from making the dataset publicly available, access may be granted to those who meet pre-specified criteria for confidential access, available at www.ices.on.ca/DAS accessed on 21 June 2022, (email: das@ices.on.ca). The full dataset creation plan and underlying analytic code are available from the authors upon request, understanding that the computer programs may rely upon coding templates or macros that are unique to ICES and are therefore either inaccessible or may require modification.

## Appendix A. Literature Search Strategy and Results (7 October 2022)

Ovid MEDLINE: Epub Ahead of Print, In-Process & Other Non-Indexed Citations, Ovid MEDLINE® Daily and Ovid MEDLINE® <1946-Present>

1 exp Neoplasms/	3743346
2 ("cancer" or malignan* or oncolog*).ti,ab.	2,504,728
3 COVID-19/ or exp COVID-19 Testing/ or COVID-19 Vaccines/ or SARS-CoV-2/	192,823
4 (coronavirus/ or betacoronavirus/ or coronavirus infections/) and (disease outbreaks/ or epidemics/ or pandemics/)	40,181
5 (nCoV* or 2019nCoV or 19nCoV or COVID19* or COVID or SARS-COV-2 or SARSCOV-2 or SARS-COV2 or SARSCOV2 or SARS coronavirus 2 or Severe Acute Respiratory Syndrome Coronavirus 2 or Severe Acute Respiratory Syndrome Corona Virus 2).ti,ab,kf,nm,ot,ox,rx,px.	290,401
6 ((new or novel or "19" or "2019" or Wuhan or Hubei or China or Chinese) adj3 (coronavirus* or corona virus* or betacoronavirus* or CoV or HCoV)).ti,ab,kf,ot.	78,800
7 (longCOVID* or postCOVID* or postcoronavirus* or postSARS*).ti,ab,kf,ot.	57
8 ((coronavirus* or corona virus* or betacoronavirus*) adj3 (pandemic* or epidemic* or outbreak* or crisis)).ti,ab,kf,ot.	13,780
9 ((Wuhan or Hubei) adj5 pneumonia).ti,ab,kf,ot.	409
10 3 or 4 or 5 or 6 or 7 or 8 or 9	302,333
11 ("new" or "incident" or diagnos* or "first" or initial*).ti,ab.	8,437,359
12 1 or 2 or 11	11,391,516
13 General Surgery/	40,392
14 ("cancer surgery" or "surgical resection" or cancer resection or surger*).ti,ab.	1,368,381
15 13 or 14	1,393,863
16 (chemo* or "systemic therapy" or radiation or radiotherap* or "hormonal therapy" or neoadjuvant or endocrine therapy).ti,ab.	1,204,385
17 10 and 12 and 15 and 16	386
18 limit 17 to (English language and humans and yr="2019-Current")	243

A comprehensive literature search for peer-reviewed English-language journal articles published between 1 Janaury 2019 and 7 October 2022 was performed on the MEDLINE database. The search yielded 243 studies, of which 238 were unique. Title/abstract screening identified 80 studies that were eligible for full-text assessment. A total of 75 studies were excluded for the following reasons: being a commentary/editorial (*n* = 9), being irrelevant to the COVID-19 pandemic (*n* = 8), being a review (*n* = 4), did not present any outcomes related to the continuum of cancer care (*n* = 53), or did not focus on newly diagnosed cancer patients (*n* = 1). These exclusions resulted in 5 studies in the final review.
